# Stress radiography of medial knee instability provides a reliable correlation with the severity of injury and medial joint space opening—A robotic biomechanical cadaveric study

**DOI:** 10.1002/ksa.12594

**Published:** 2025-01-22

**Authors:** Thorben Briese, Matthias Holz, Christian Peez, Michael J. Raschke, Adrian Deichsel, Elmar Herbst, Mirco Herbort, Christoph Kittl

**Affiliations:** ^1^ Department of Trauma, Hand and Reconstructive Surgery University Hospital Muenster Muenster Germany; ^2^ OCM Orthopedic Surgery Munich Munich Germany

**Keywords:** medial collateral ligament, medial gaping, medial instability, stress x‐ray, valgus stress

## Abstract

**Purpose:**

The medial collateral ligament (MCL), and posterior oblique ligament (POL) are the primary valgus stabilisers of the knee, and clinical examinations in grading valgus instability can be inherently subjective. Stress radiography of medial‐sided knee injuries provides objective diagnosis and was analysed in this study. We hypothesised that (1) medial joint space opening would increase cutting the superficial MCL (sMCL), POL and anterior cruciate ligament (ACL); (2) isolated deep MCL (dMCL) injury would not increase medial joint space opening; (3) medial joint space opening would increase at higher flexion angles.

**Study Design:**

Controlled laboratory study.

**Methods:**

Ten human cadaveric knees were dissected, preserving ligamentous structures, muscles and fascia. The femur was secured, and the tibia was attached to the six‐degree‐of‐freedom robot. A 10 Nm valgus torque was applied at 0°–45° of flexion and anterior‐posterior (a.p.) radiographs were taken. Sequential sectioning was performed on the dMCL, sMCL, POL and ACL. Medial joint space opening was measured on a.p. radiographs (midpoint technique). Statistical analysis was conducted using a mixed model with post hoc correction (*p* < 0.05). Intra‐ and interobserver reliability was assessed by calculating the intraclass correlation coefficient (ICC).

**Results:**

Medial joint space opening significantly increased with cutting state (*p* < 0.0001) and flexion angle (*p* < 0.0001). Although isolated dMCL injury did not significantly increase medial joint space opening, sMCL resection gradually increased joint space opening 3.2 ± 1.9 to 6.9 ± 2.7 mm (*p* = 0.039) between 0° and 45° knee flexion. Following POL deficiency, medial joint space opening further increased 6.4 ± 2.7 to 11.4 ± 6.2 mm between 0° and 45° knee flexion (*p* = 0.0035). A combined injury (dMCL/sMCL/POL/ACL) increased medial joint space opening 12.0 ± 4.9 to 21.8 ± 7.9 mm (*p* < 0.0001) between 0° and 45° knee flexion, compared to the intact state. The intraobserver ICC was 0.995 and the interobserver ICC was 0.955 showing excellent intra‐ and interobserver reliability.

**Conclusion:**

Deficiency of the medial stabilisers of the knee increased medial joint space opening in stress radiography, whereas isolated dMCL deficiency did not significantly affect valgus gapping. This study demonstrated a good concordance between valgus stress radiography and clinical scores (International Knee Documentation Committee and Hughston). Our findings support performing valgus stress tests at 0° and at least 20° of flexion.

**Level of Evidence:**

There is no level of evidence as this study was an experimental laboratory study.

**Abbreviations**
ACL: anterior cruciate ligament
a.p.: anterior‐posterior
C: celsius
cm: centimetre
dMCL: deep medial collateral ligament
ICC: intraclass correlation coefficient
IKDC: International Knee Documentation Committee
IRB: institutional review board
MCL: medial collateral ligament
mm: millimetre
N: newton
Nm: newton‐metre
n.s.: not significant
POL: posterior oblique ligament
SD: standard deviation
sMCL: superficial medial collateral ligament
UFS: universal force sensor

## INTRODUCTION

In knee ligament injuries, the anterior cruciate ligament (ACL) and the medial stabilisers are most frequently affected [6, 26, 33], with injuries to the medial collateral ligament (MCL) accounting for 7.9% of all injuries [29]. The superficial MCL (sMCL) and posterior oblique ligament (POL) are primary restraints against valgus stress and anteromedial rotation, while the ACL serves as a secondary restraint [5, 10, 15, 18, 19, 35, 39, 40]. Combined medial injuries (ACL, MCL and POL) can lead to chronic valgus instability and pain [22, 23] compromising function and resulting in higher failure rates of ACL reconstructions [1, 2, 5, 36].

Decision‐making for surgical treatment typically indicated for grade III injuries (>10 mm medial opening according to the Hughston classification), often relies on subjective estimation [3, 14, 21, 34]. However, clinical examination and evaluation of medial joint opening can be challenging being dependent on the examiner's skill. The diagnostic accuracy of MRI for identifying medial injuries is high [32, 43] but fails grading instability. Valgus stress radiographs may provide a more objective assessment, potentially enhancing decision‐making in cases of medial knee instability. Previous biomechanical studies on stress radiography have indicated that existing classifications may underestimate the severity of medial instability [25] and did not perform valgus gapping above 20° knee flexion angle. It has also been shown that the deep MCL (dMCL) is an important restraint to external rotation and anteromedial rotatory instability [4]. Rotational instabilities are often difficult to diagnose clinically [20], so the ability to identify an isolated dMCL injury through stress radiography would be valuable.

Therefore, the aim of this study was to investigate medial joint space opening in stress radiography following sequential cutting of the medial stabilisers at various flexion angles. We hypothesised that: (1) medial joint space opening would be significantly increased by the sectioning of the sMCL, POL and ACL; (2) an isolated deficiency to the dMCL would not significantly increase medial joint space opening and (3) medial joint space opening would increase with higher flexion angles.

## MATERIALS AND METHODS

Ten fresh‐frozen cadaveric knee specimens from the anatomical department of the University of Luebeck (Germany) were included in the study. The exclusion criteria included prior surgery, high‐grade osteoarthritis (Kellgren‐Lawrence Grade ≥ III) and ligamentous instability. Prior to testing, ligamentous integrity was confirmed through a clinical examination. High‐grade osteoarthritis was ruled out after testing was performed by exarticulation of the knee joints. All 10 specimens could be included in the final analysis. The specimens were stored at −20°C and thawed for 24 h at room temperature, prior to preparation and testing. Before testing, the knees were flexed 10 times to prevent tissue hysteresis [30].

### Specimen preparation

**Figure 1:**
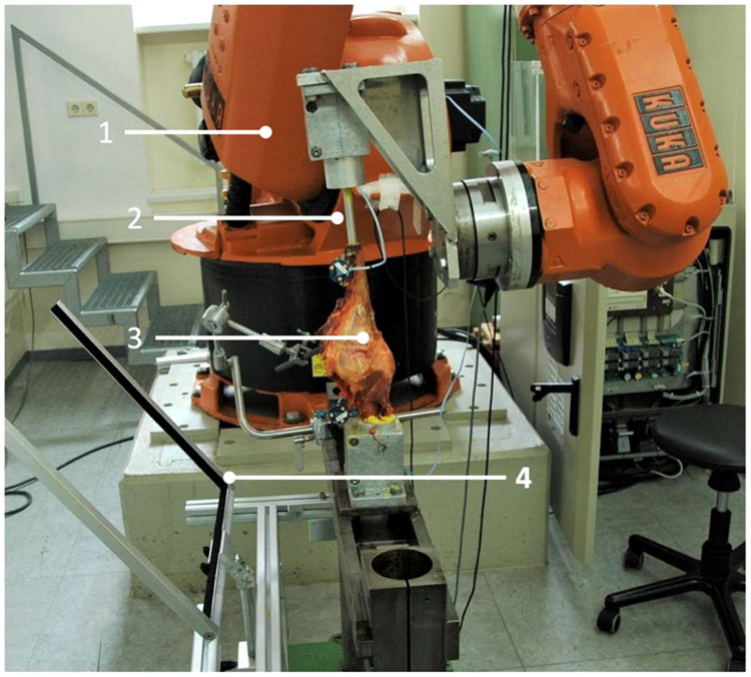
Left knee (3) mounted upside‐down in the robotic test setup (1). To avoid obstruction with the moving arm, the knee was extended using a tibial nail (2). After applying valgus stress, an anterior‐posterior radiograph could be performed using a detector screen, which was mounted in a custom‐made fixture (4). The construct could be angulated to account for knee alignment in the robot and flexion angle.

After the resection of the skin and subcutaneous fat, the femur and tibia were cut 20 cm above and below the joint line. Muscles were removed approximately 8 cm proximal and distal to the knee joint line. The fibula was sectioned at the same level as the resected muscles and secured to the proximal tibia using a 4 × 40 mm cortical screw. To facilitate unobstructed anterior‐posterior radiographs during robotic testing, a 340 mm aluminium rod with a 10 mm diameter was inserted into the intramedullary canal of the tibia, embedded with synthetic resin and two 4 × 40 mm cortical screws. This rod was then secured within an aluminium cylinder and synthetic resin. The tibial aluminium pot was fixed to the moving arm of the robot, ensuring that the posterior condyles of the femur were aligned parallel to the stationary base of the robot. Finally, the femur was secured into an aluminium pot with synthetic resin and fixated to the stationary base (Figure 1).

### Ethics approval

The experiments were performed with the approval of the Institutional Review Board of the University of Muenster (IRB reference number 2014‐421‐f‐N).

### Test setup

Measurements were performed using a robotic test setup, which has already been established in several biomechanical studies [7, 11, 38, 44]. The six‐degree of freedom robotic system (Type KR125; Kuka robot GmbH) ensured free movement of the tibia, while forces/torques and the flexion‐extension passive path of the knees were recorded via a Universal Force Sensor (UFS) (Ametech Robotics GmbH). This system operated in a force‐controlled mode via a force feedback loop from the UFS to the robot [7, 11, 38, 44], guaranteeing exact force/torque application to the specimens. The robot has a maximum load capacity of 2450 N and a repeatability of ±0.2 mm. The resolution of the force/torque sensor was 0.25 N and 0.05 Nm for forces and torques, respectively.

**Figure 2:**
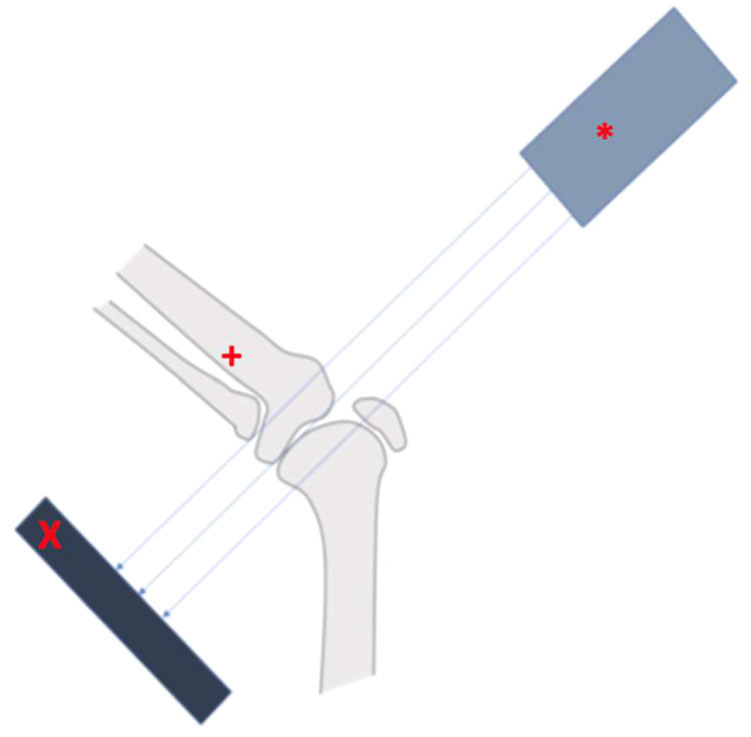
Schematic illustration of the x‐ray test setup. The x‐ray source was always positioned orthogonal to the detector and slope neutral to the medial tibial plateau; x, detector; +, specimen in upside down position; *, x‐ray source.

The test setup included an x‐ray detector and an x‐ray source, which were positioned 50 cm behind and 50 cm in front of the knee specimen, respectively (X‐Ray‐Source: AJEX9020H, Ajex Meditech Ltd.; X‐Ray‐Detector: 1717SGC, Rayence) (Figure 2). A 32 mm diameter x‐ray reference ball was used in all radiographs. For standardised repetition of the radiographs, the x‐ray detector and source were mounted in a custom‐made fixture, which could be angulated to always create a true anterior‐posterior radiograph of the knee.

### Testing protocol

Once the knee was secured in the robot, its position was manually adjusted to achieve full extension by minimising forces and torques across all axes. The robot then initiated the passive path, flexing the knee to 45° in 1° increments while automatically minimising forces and torques in all axes except for the flexion/extension axis. The robot proceeded to flex the knee to 0°, 10°, 20°, 30° and 45°, applying a valgus torque of 10 Nm at each position. This valgus torque was maintained until the anterior‐posterior radiograph was taken.

**Figure 3:**
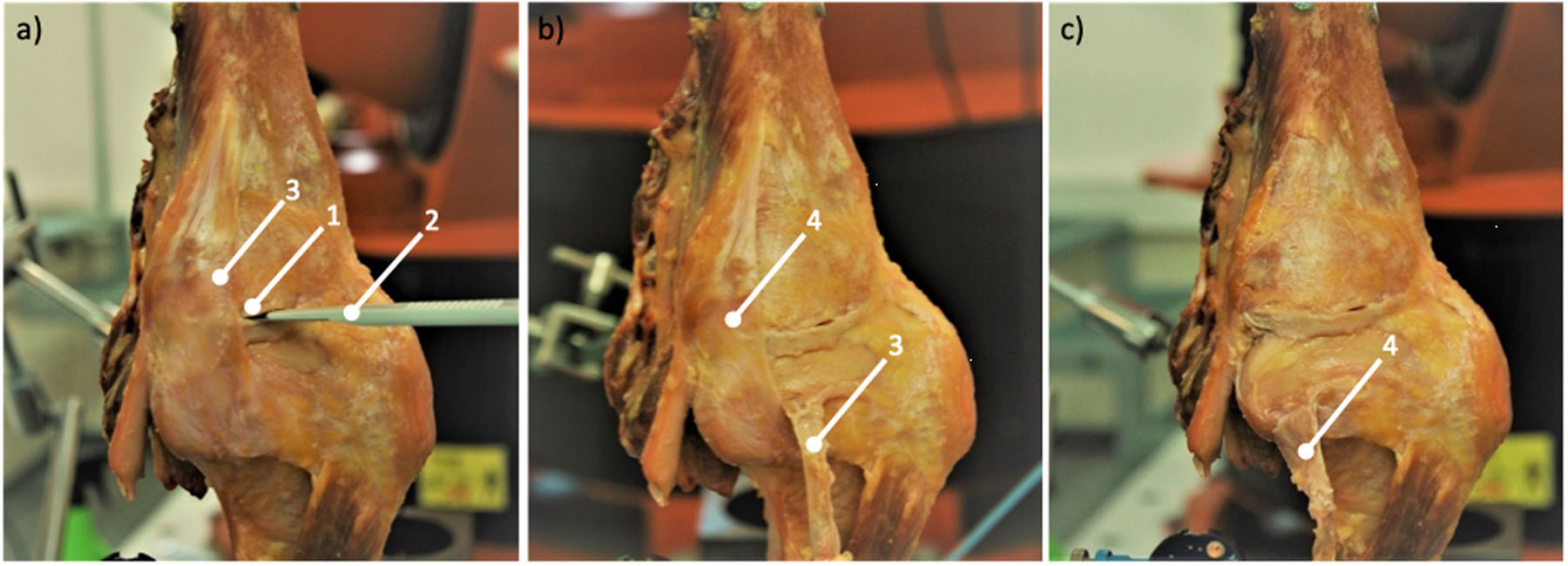
Left knee mounted upside‐down in the robotic test setup. (a) Cutting the deep medial collateral ligament (1) using a scalpel (2), which was slid deep to the superficial medial collateral ligament (3). (b) The superficial medial collateral ligament (3) was identified as all the fibres running parallel from the medial femoral epicondyle to its tibial insertion where it was resected. The posterior oblique ligament (4) is intact. (c) As the final cutting step, the posterior oblique ligament (4) was resected.

This was performed three times for each flexion and cutting state. The following structures were resected sequentially (Figure 3):

(1) The meniscofemoral and meniscotibial portion of the dMCL was cut deep to the sMCL. Therefore, a scalpel was slid above and below the meniscus and the corresponding portion of the dMCL was cut until the dMCL blended with the posteromedial capsule.
(2) The sMCL was defined as all fibres running parallel from the medial femoral epicondyle distally. These fibres were completely resected, including their portion at the joint line.
(3) The superficial, central and capsular arms of the POL were resected completely.
(4) The ACL was cut midsubstance.

The anteromedial retinaculum and the anteromedial capsule were resected before the experiments started because it is known from previous studies that these structures only play a minor role in influencing valgus rotation [4, 19].

### Radiographic measurements

**Figure 4:**
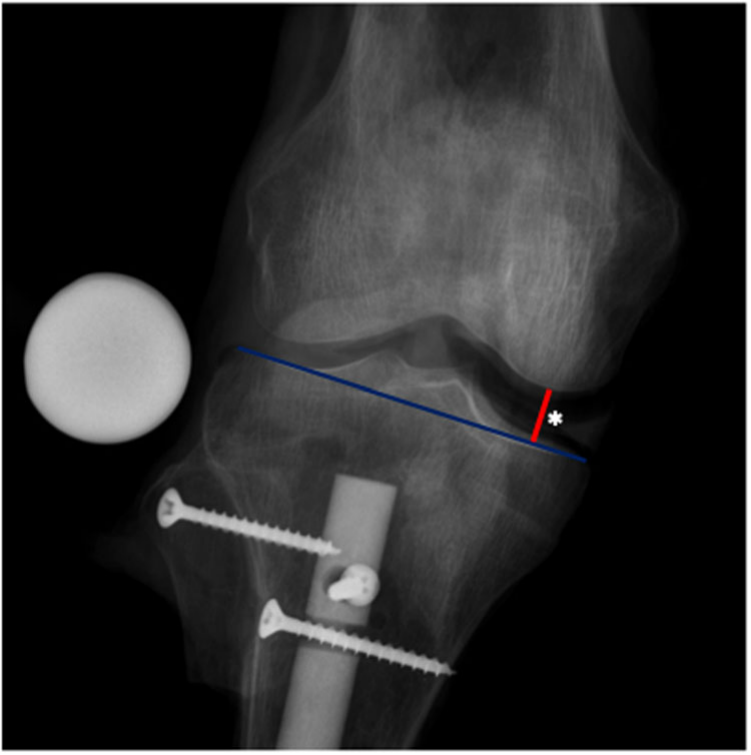
Anterior‐posterior stress radiograph (10 Nm valgus torque) of a right human cadaveric knee in 0° flexion with sectioned deep and superficial medial collateral ligament. Medial joint space opening was measured using the midpoint technique [31]. Therefore, a line along the tibial plateau was drawn (blue). Another line (red line) was drawn perpendicular to the tibial plateau between the most convex point of the medial femoral condyle and the blue line representing the medial joint space opening (*).

The medial joint space opening was measured in each radiograph using the midpoint technique [31] according to previous studies [25] (Figure 4). A 32 mm diameter x‐ray reference ball was used. The mean of the three measurements of each state and flexion angle was used for the final analysis. The radiographs were analysed using a DICOM Viewer (OsiriX MD v.14.0 Dicom, Pixmeo SARL). The measurements were recorded to the nearest 0.1 mm, consistent with the accuracy of the DICOM Viewer. Two experienced orthopaedic surgeons independently performed the radiographic measurements. For intraobserver reliability, rater one (T. B.) performed measurements twice, while for interobserver reliability, two raters (T. B.; C. K.) each performed measurements once.

### Data and statistical analysis

An a‐priori power analysis was performed using G*Power‐2 software (University Düsseldorf) [13]. Based on means and standard deviations from a prior study (1.7 ± 1 mm) [25], it was assumed that a sample size of a minimum of eight would allow the identification of changes of the medial joint space opening of 1.7 mm, with a standard deviation of 1 mm (effect size/Cohen's *d* = 1.88), with 90% power, at the significance level of *p* < 0.05. A *p* ≤ 0.05 was considered statistically significant. A mixed model with Tukey's post hoc correction was used for statistical analysis that was performed using Graph Pad Prism 10 (Version 10.0.0). Intra‐ and interobserver reliability of the measurements was determined by calculating the intraclass correlation coefficient (ICC) in Excel (Microsoft). An ICC of 0.60–0.74 was considered good, and 0.75–1.00 was considered excellent reliability [16].

## RESULTS

### Effect of grade of medial injury on medial joint space opening

Medial joint space opening significantly increased with the grade of medial injury (main effect, ‘F (4, 220) = 11,89’, *p* < 0.0001) meaning that an increased medial instability caused more medial joint space opening.

### Effect of flexion angle on medial joint space opening

Medial joint space opening significantly increased with increased flexion angle (0°–45°) (main effect, ‘F (4, 220) = 174,6’, *p* < 0.0001), meaning that medial joint space opening for the same cutting state increased with higher flexion angles.

### Change of medial joint space opening depending on medial instability

**Figure 5:**
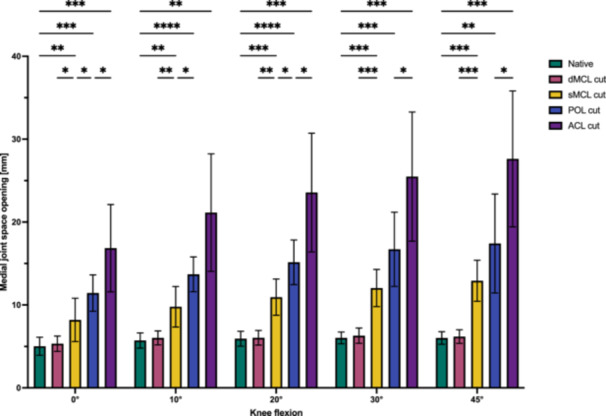
Medial joint space opening depending on cutting state between 0° and 45° flexion. *p < 0.05; **p < 0.01; ***p < 0.001); deep medial collateral ligament (dMCL), superficial medial collateral ligament (sMCL), posterior oblique ligament (POL) and anterior cruciate ligament (ACL).

A simulated dMCL injury showed no significant increase (0.3 ± 0.5–0.2 ± 0.3 mm) in medial joint space opening in all tested flexion angles, compared with the intact state (n.s.). After completely resecting the sMCL, the medial joint opening gradually increased 3.2 ± 1.9 mm (0°, *p* = 0.007), 4.1 ± 2.0 mm (10°, *p* = 0.002), 5.0 ± 2.1 mm (20°, *p* = 0.0005), 6.0 ± 2.2 mm (30°, *p* = 0.0001) and 6.9 ± 2.7 mm (45°, *p* = 0.0002), compared to the intact state. After resecting the POL, the medial joint space opening further increased 6.4 ± 2.7 mm (0°, *p* = 0.0003), 8.0 ± 2.6 mm (10°, *p *< 0.0001), 9.2 ± 3.1 mm (20°, *p* < 0.0001), 10.7 ± 4.6 mm (30°, *p* = 0.0004) and 11.4 ± 6.2 mm (45°, *p* = 0.002), compared to the intact state. A simulated combined dMCL/sMCL/POL/ACL injury had the highest increase in medial joint space opening, compared to the intact state with 12.0 ± 4.9 mm (0°, *p* = 0.0008), 15.6 ± 6.9 mm (10°, *p* = 0.0012), 17.8 ± 6.6 mm (20°, *p* = 0.0005), 19.6 ± 7.1 mm (30°, *p* = 0.0004) and 21.8 ± 7.9 mm (45°, *p* = 0.0003). See Table 1 and Figure 5 for additional information.

**Table 1:** Absolute values of medial joint space opening in mm depending on the severity of medial injury and in the different flexion angles compared to the intact state; change of medial joint space opening in mm compared to the intact state.

Average medial joint space opening compared with the intact knee (mm ± SD)						
**Cutting state**		0°	10°	20°	30°	45°
**Intact**	Absolute	5.0 ± 1.1	5.7 ± 0.9	5.9 ± 0.9	6.0 ± 0.7	6.0 ± 0.8
**dMCL**	Absolute	5.3 ± 0.9	6.0 ± 0.8	6.0 ± 0.9	6.3 ± 0.9	6.2 ± 0.8
	*Change*	*0.3* ± *0.5*	*0.3* ± *0.4*	*0.1* ± *0.7*	*0.3* ± *0.8*	*0.2* ± *0.3*
**sMCL**	Absolute	8.2 ± 2.3^a^	9.8 ± 2.2^a^	10.9 ± 2.0^a^	12.0 ± 2.1^a^	12.9 ± 2.5^a^
	*Change*	*3.2* ± *1.9* a	*4.1* ± *2.0* a	*5.0* ± *2.1* a	*6.0* ± *2.2* a	*6.9* ± *2.7* a
**POL**	Absolute	11.4 ± 2.2^a^	13.7 ± 2.1^a^	15.2 ± 2.6^a^	16.7 ± 4.3	17.4 ± 5.8
	*Change*	*6.4* ± *2.7* a	*8.0* ± *2.6* a	*9.2* ± *3.1* a	*10.7* ± *4.6*	*11.4* ± *6.2*
**ACL**	Absolute	16.9 ± 5.0^a^	21.1 ± 6.8	23.6 ± 6.6^a^	25.5 ± 7.2^a^	27.6 ± 7.8^a^
	*Change*	*12.0* ± *4.9* a	*15.6* ± *6.9*	*17.8* ± *6.6* a	*19.6* ± *7.1* a	*21.8* ± *7.9* a

Abbreviations: ACL, anterior cruciate ligament; dMCL, deep medial collateral ligament; mm, millimetre; POL, posterior oblique ligament; SD, standard deviation; sMCL, superficial medial collateral ligament.

^a^ Significant change of the medial joint space opening compared with the previous sectioned state.

### Intra‐ and Interobserver reliability

The intraobserver ICC was 0.995 and the interobserver ICC was 0.955 showing excellent intra‐ and interobserver reliability.

## DISCUSSION

The main finding of the present study was that, based on our hypothesis, the medial joint space opening gradually increased with medial instability. Furthermore, medial joint space opening increased with flexion angle. For example, a clinically relevant grade III sMCL and combined dMCL instability [17, 42] presented a 3.2 mm medial joint space opening at full extension (side‐to‐side difference), which increased to 6 mm at 30° knee flexion. This increased up to 11.9 and 19.2 mm in a knee dislocation type of injury (dMCL/sMCL/POL/ACL). These findings indicate that considerable medial gapping at 0° of flexion should raise suspicion for multiligament injuries. Another important finding of the present study was that an isolated dMCL injury had no effect on medial joint space opening, confirming the results of a previous study concerning dMCL injury and valgus stress [40]. This suggests that valgus stress radiography does not allow for the identification of isolated anteromedial rotational instability.

Similar to the present study, a previous in‐vitro study [25] investigated the medial gapping in 0° and 20° knee flexion in correlation with the medial injury in stress radiographs [25], whereas different knee flexion angles (10°, 30° and 45°) were missing. In their study, the 10 Nm valgus force was applied manually by clinicians using a hand dynamometer force sensor for feedback, rather than a robotic setup, which was used in our present study. However, their order of ligament sectioning differed, as the sMCL was always cut first; therefore, the influence of the dMCL could not be analysed. This was the reason we performed the sectioning of the dMCL first, to analyse its influence on valgus gapping in stress radiography for the first time. Despite these differences, their findings indicated that complete sectioning of both the sMCL and dMCL at 20° of flexion resulted in a medial gapping of 13.3 ± 2.6 mm, while our data showed less gapping of 10.94 ± 2.19 mm at 20° and 12.04 ± 2.24 mm at 30° of flexion, respectively. They also identified the POL and ACL as significant restraints against valgus stress [25]. Ciba et al. [9] conducted an in‐vitro biomechanical study examining medial gapping during 30° valgus stress in MRIs. They reported an increase in medial gapping to 11.0 ± 1.4 mm in cases of isolated sMCL injury, which aligns with our findings and correlates with an abnormal International Knee Documentation Committee (IKDC) and a Hughston Grade III classification. After an additional ACL deficiency, medial gapping increased to 12.1 ± 1.4 mm. Importantly, the POL remained intact in their study, making only the sMCL values comparable.

The clinical relevance of this study lies in its demonstration that stress radiography aids in identifying instabilities, which may require surgery. Concerning clinical applicability, we tried to correlate our findings with the IKDC and Hughston classification, as shown in Table 2, by comparing our values of medial joint space opening for each cutting state with the cut‐off values of the IKDC and Hughston classification. This showed that an increased medial gapping in full extension (>10 mm) indicated an additional POL injury, being important for surgical decision‐making [3, 14, 21, 34]. Conversely, an increased medial gapping in at least 20° flexion (>10 mm; with less increase in 0°) should be suspected for an isolated sMCL injury, which may, therefore, be treated conservatively [8, 12, 27, 37, 41]. Our data support the clinical practice of applying valgus force at 0° and higher degrees of flexion, with a preference for 0° and at least 20° of flexion. The importance of stress radiography is reinforced by a systematic review conducted by Mabrouk et al., which highlighted the accuracy of stress radiography in objectively diagnosing ligamentous injuries [28], which is also shown by our high inter‐ and intraclass ICC.

**Table 2:** Concordance of our results with IKDC, and Hughston classification.

Flexion	Cutting state	IKDC	Hughston
0°	**dMCL cut**	Normal	Grade II
	**sMCL cut**	Nearly normal	Grade II
	**POL cut**	Abnormal	Grade III
	**ACL cut**	Severely abnormal	Grade III
10°	**dMCL cut**	Normal	Grade II
	**sMCL cut**	Nearly normal	Grade II
	**POL cut**	Abnormal	Grade III
	**ACL cut**	Severely abnormal	Grade III
20°	**dMCL cut**	Normal	Grade II
	**sMCL cut**	Abnormal	Grade III
	**POL cut**	Abnormal	Grade III
	**ACL cut**	Severely abnormal	Grade III
30°	**dMCL cut**	Normal	Grade II
	**sMCL cut**	Abnormal	Grade III
	**POL cut**	Severely abnormal	Grade III
	**ACL cut**	Severely abnormal	Grade III
45°	**dMCL cut**	Normal	Grade II
	**sMCL cut**	Abnormal	Grade III
	**POL cut**	Severely abnormal	Grade III
	**ACL cut**	Severely abnormal	Grade III

*Note*: IKDC describes the side‐to‐side difference with an intact state, and Hughston describes the absolute values of medial joint space opening. IKDC: 0–2 mm = normal, 2–5 mm = nearly normal, 5–10 mm = abnormal, >10 mm = severely abnormal; Hughston: <5 mm = Grade I, 5–10 mm = Grade II, >10 mm = Grade III. Intact state (not shown) already showed a Hughston Grade I–II instability.

Abbreviations: ACL, anterior cruciate ligament; dMCL, deep medial collateral ligament; IKDC, International Knee Documentation Committee; POL, posterior oblique ligament; sMCL, superficial medial collateral ligament.

This study had limitations inherent to in‐vitro biomechanical studies. The age of the used specimens is higher compared to most patients seen in the clinical setting, which suffer from these injuries. The created lesions only mimic the injury pattern of acute time‐zero injuries to the medial structures and in vivo; even in a short time frame, the results could differ due to healing and scaring of the structures. Furthermore, the present study only investigated the effect of the passive stabilisers on the medial side of the knee. Influence of the active stabilisers of the medial side such as the semimembranosus [24]. Another limitation is that the study employed a robotic test setup, which applied a standardised torque of 10 Nm, whereas, in clinical practice, valgus stress is typically applied using a stress device (e.g., Telos device) or manually by clinicians. Additionally, the measurements were only recorded to the nearest 0.1 mm. Furthermore, this study involved complete resection of the respective structures rather than making specific cuts (e.g., intraligamentous or at the femoral/tibial attachments). This approach may be an important factor, as evidenced by differing results in the investigation by LaPrade et al. [25]. Furthermore, we did not randomise the cutting states to differentiate between isolated injuries to the sMCL, POL or ACL, but a comparable study on stress radiography for medial knee injury also only randomised the tibial or femoral insertions of the MCL rather than the ligaments itself [25].

## CONCLUSION

Deficiency of the medial stabilisers of the knee increased medial joint space opening in stress radiography, whereas isolated dMCL deficiency did not significantly affect valgus gapping. This study demonstrated a good concordance between valgus stress radiography and clinical scores (IKDC and Hughston). Our findings support performing valgus stress tests at 0° and at least 20° of flexion.

## AUTHOR CONTRIBUTIONS

**Thorben Briese**: Conception and design; testing and data acquisition; statistical analysis; writing. **Matthias Holz**: Testing and data acquisition; writing; statistical analysis. **Christian Peez**: Internal review; statistical analysis. **Michael J. Raschke**: Internal review. **Adrian Deichsel**: Internal review. **Elmar Herbst**: Internal review. **Mirco Herbort**: Internal review; conception and design. **Christoph Kittl**: Conception and design; testing and data acquisition; statistical analysis; writing.

## CONFLICT OF INTEREST STATEMENT

Elmar Herbst is Deputy Editor‐in‐Chief for the Knee Surgery, Sports Traumatology and Arthroscopy (KSSTA). Adrian Deichsel is the Web Editor for the Knee Surgery, Sports Traumatology and Arthroscopy (KSSTA). The remaining authors declare no conflicts of interest.

## ETHICS STATEMENT

The specimens were dissected and biomechanically tested under the approval of the Institutional Ethics Committee of the University of Muenster (IRB reference number 2014‐421‐f‐N).

## Data Availability

Data are available from the corresponding author upon reasonable request.

## References

[1] Alm L , Drenck TC , Frings J , Krause M , Korthaus A , Krukenberg A , et al. Lower failure rates and improved patient outcome due to reconstruction of the MCL and revision ACL reconstruction in chronic medial knee instability. Orthop J Sports Med. 2021;9:2325967121989312. 33796589 10.1177/2325967121989312 PMC7968026

[2] Alm L , Krause M , Frosch KH , Akoto R . Preoperative medial knee instability is an underestimated risk factor for failure of revision ACL reconstruction. Knee Surg Sports Traumatol Arthrosc. 2020;28:2458–2467. 32621041 10.1007/s00167-020-06133-y PMC7429520

[3] Rachun A . Standard nomenclature of athletic injuries. Chicago: American Medical Association; 1968.

[4] Ball S , Stephen JM , El‐Daou H , Williams A , Amis AA . The medial ligaments and the ACL restrain anteromedial laxity of the knee. Knee Surg Sports Traumatol Arthrosc. 2020;28:3700–3708. 32504158 10.1007/s00167-020-06084-4 PMC7669770

[5] Battaglia MJ , Lenhoff MW , Ehteshami JR , Lyman S , Provencher MT , Wickiewicz TL , et al. Medial collateral ligament injuries and subsequent load on the anterior cruciate ligament: a biomechanical evaluation in a cadaveric model. Am J Sports Med. 2009;37:305–311. 19098154 10.1177/0363546508324969

[6] Bollen S . Ligament injuries of the knee‐‐limping forward? Br J Sports Med. 1998;32:82–84. 9562174 10.1136/bjsm.32.1.82 PMC1756072

[7] Carlin GJ , Livesay GA , Harner CD , Ishibashi Y , Kim HS , Woo SLY . In‐situ forces in the human posterior cruciate ligament in response to posterior tibial loading. Ann Biomed Eng. 1996;24:193–197. 8678351 10.1007/BF02667348

[8] Chahla J , Kunze KN , LaPrade RF , Getgood A , Cohen M , Gelber P , et al. The posteromedial corner of the knee: an international expert consensus statement on diagnosis, classification, treatment, and rehabilitation. Knee Surg Sports Traumatol Arthrosc. 2021;29:2976–2986. 33104867 10.1007/s00167-020-06336-3 PMC7586411

[9] Ciba M , Winkelmeyer EM , Schock J , Schad P , Kotowski N , Nolte T , et al. Comprehensive assessment of medial knee joint instability by valgus stress MRI. Diagnostics. 2021;11(8):1433. 34441368 10.3390/diagnostics11081433 PMC8392372

[10] Deichsel A , Peez C , Raschke MJ , Albert A , Herbort M , Kittl C , et al. A flat reconstruction of the medial collateral ligament and anteromedial structures restores native knee kinematics: a biomechanical robotic investigation. Am J Sports Med. 2024;52:3306–3313. 39360333 10.1177/03635465241280984 PMC11542325

[11] Diermann N , Schumacher T , Schanz S , Raschke MJ , Petersen W , Zantop T . Rotational instability of the knee: Internal tibial rotation under a simulated pivot shift test. Arch Orthop Trauma Surg. 2008;129:353–358. 18594847 10.1007/s00402-008-0681-z

[12] Engebretsen L , Lind M . Anteromedial rotatory laxity. Knee Surg Sports Traumatol Arthrosc. 2015;23:2797–2804. 26085190 10.1007/s00167-015-3675-8

[13] Faul F , Erdfelder E , Buchner A , Lang A‐G . Statistical power analyses using G*Power 3.1: tests for correlation and regression analyses. Behav Res Methods. 2009;41:1149–1160. 19897823 10.3758/BRM.41.4.1149

[14] Fetto JF , Marshall JL . Medial collateral ligament injuries of the knee: a rationale for treatment. Clin Orthop Relat Res. 1978;132:206–218. 679543

[15] Fischer RA , Arms SW , Johnson RJ , Pope MH . The functional relationship of the posterior oblique ligament to the medial collateral ligament of the human knee. Am J Sports Med. 1985;13:390–397. 4073346 10.1177/036354658501300605

[16] Fleiss JL . Design and analysis of clinical experiments. Wiley; 2011.

[17] Grunenberg O , Gerwing M , Oeckenpöhler S , Peez C , Briese T , Glasbrenner J , et al. The anteromedial retinaculum in ACL‐injured knees: an overlooked injury? Knee Surg Sports Traumatol Arthrosc. 2024;32:881–888. 38469949 10.1002/ksa.12137

[18] Haimes JL , Wroble RR , Grood ES , Noyes FR . Role of the medial structures in the intact and anterior cruciate ligament‐deficient knee. Limits of motion in the human knee. Am J Sports Med. 1994;22:402–409. 8037282 10.1177/036354659402200317

[19] Herbst E , Muhmann RJ , Raschke MJ , Katthagen JC , Oeckenpöhler S , Wermers J , et al. The aterior fibers of the superficial MCL and the ACL restrain anteromedial rotatory instability. Am J Sports Med. 2023;51:2928–2935. 37503921 10.1177/03635465231187043

[20] Hughes JD , Rauer T , Gibbs CM , Musahl V . Diagnosis and treatment of rotatory knee instability. J Exp Orthop. 2019;6:48. 31865518 10.1186/s40634-019-0217-1 PMC6925612

[21] Hughston J , Andrews J , Cross M , Moschi A . Classification of knee ligament instabilities. Part I. The medial compartment and cruciate ligaments. J Bone Joint Surg Am. 1976;58:159–172. 1254619

[22] Jones L , Bismil Q , Alyas F , Connell D , Bell J . Persistent symptoms following non operative management in low grade MCL injury of the knee—The role of the deep MCL. Knee. 2009;16:64–68. 18938083 10.1016/j.knee.2008.09.002

[23] Kannus P . Long‐term results of conservatively treated medial collateral ligament injuries of the knee joint. Clin Orthop Relat Res. 1988;226:103–112. 3335084

[24] Kittl C , Becker DK , Raschke MJ , Müller M , Wierer G , Domnick C , et al. Dynamic restraints of the medial side of the knee: the semimembranosus corner revisited. Am J Sports Med. 2019;47:863–869. 30870030 10.1177/0363546519829384

[25] Laprade RF , Bernhardson AS , Griffith CJ , Macalena JA , Wijdicks CA . Correlation of valgus stress radiographs with medial knee ligament injuries: an in vitro biomechanical study. Am J Sports Med. 2010;38:330–338. 19966093 10.1177/0363546509349347

[26] Laprade RF , Wijdicks CA . The management of injuries to the medial side of the knee. J Orthop Sports Phys Ther. 2012;42:221–233. 22382986 10.2519/jospt.2012.3624

[27] Lundberg M , Messner K . Ten‐year prognosis of isolated and combined medial collateral ligament ruptures. A matched comparison in 40 patients using clinical and radiographic evaluations. Am J Sports Med. 1997;25:2–6. 9006684 10.1177/036354659702500102

[28] Mabrouk A , Olson CP , Tagliero AJ , Larson CM , Wulf CA , Kennedy NI , et al. Reference standards for stress radiography measurements in knee ligament injury and instability: a systematic review. Knee Surg Sports Traumatol Arthrosc. 2023;31:5721–5746. 37923947 10.1007/s00167-023-07617-3

[29] Majewski M , Susanne H , Klaus S . Epidemiology of athletic knee injuries: a 10‐year study. Knee. 2006;13:184–188. 16603363 10.1016/j.knee.2006.01.005

[30] Martin RB , Burr DB , Sharkey NA , Fyhrie DP . Skeletal tissue mechanics. 190. New York: Springer; 1998.

[31] Mehta N , Duryea J , Badger GJ , Akelman MR , Jones MH , Spindler KP , et al. Comparison of 2 radiographic techniques for measurement of tibiofemoral joint space width. Orthop J Sports Med. 2017;5:2325967117728675. 28989937 10.1177/2325967117728675 PMC5624356

[32] Meyer P , Reiter A , Akoto R , Steadman J , Pagenstert G , Frosch KH , et al. Imaging of the medial collateral ligament of the knee: a systematic review. Arch Orthop Trauma Surg. 2022;142:3721–3736. 34628563 10.1007/s00402-021-04200-8 PMC9596543

[33] Miyasaka K . The incidence of knee ligament injuries in the general population. Am J Knee Surg. 1991;1:43–48.

[34] Phisitkul P , James SL , Wolf BR , Amendola A . MCL injuries of the knee: current concepts review. Iowa Orthop J. 2006;26:77–90. 16789454 PMC1888587

[35] Robinson JR , Sanchez‐Ballester J , Bull AMJ , Thomas RWM , Amis AA . The posteromedial corner revisited. An anatomical description of the passive restraining structures of the medial aspect of the human knee. J Bone Joint Surg Br. 2004;86:674–681. 15274262 10.1302/0301-620x.86b5.14853

[36] Svantesson E , Hamrin Senorski E , Alentorn‐Geli E , Westin O , Sundemo D , Grassi A , et al. Increased risk of ACL revision with non‐surgical treatment of a concomitant medial collateral ligament injury: a study on 19,457 patients from the Swedish National Knee Ligament Registry. Knee Surg Sports Traumatol Arthrosc. 2019;27:2450–2459. 30374568 10.1007/s00167-018-5237-3 PMC6656795

[37] Svantesson J , Piussi R , Weissglas E , Svantesson E , Horvath A , Börjesson E , et al. Shedding light on the non‐operative treatment of the forgotten side of the knee: rehabilitation of medial collateral ligament injuries‐a systematic review. BMJ Open Sport Exerc Med. 2024;10:e001750. 10.1136/bmjsem-2023-001750 PMC11202733 38933372

[38] Tachibana Y , Mae T , Fujie H , Shino K , Ohori T , Yoshikawa H , et al. Effect of radial meniscal tear on in situ forces of meniscus and tibiofemoral relationship. Knee Surg Sports Traumatol Arthrosc. 2017;25:355–361. 28012003 10.1007/s00167-016-4395-4

[39] Warren LF , Marshall JL . The supporting structures and layers on the medial side of the knee: an anatomical analysis. J Bone Joint Surg Am. 1979;61:56–62. 759437

[40] Wierer G , Milinkovic D , Robinson JR , Raschke MJ , Weiler A , Fink C , et al. The superficial medial collateral ligament is the major restraint to anteromedial instability of the knee. Knee Surg Sports Traumatol Arthrosc. 2021;29:405–416. 32277264 10.1007/s00167-020-05947-0

[41] Wijdicks CA , Griffith CJ , Johansen S , Engebretsen L , LaPrade RF . Injuries to the medial collateral ligament and associated medial structures of the knee. J Bone Joint Surg Am. 2010;92:1266–1280. 20439679 10.2106/JBJS.I.01229

[42] Willinger L , Balendra G , Pai V , Lee J , Mitchell A , Jones M , et al. High incidence of superficial and deep medial collateral ligament injuries in ‘isolated’ anterior cruciate ligament ruptures: a long overlooked injury. Knee Surg Sports Traumatol Arthrosc. 2022;30:167–175. 33661325 10.1007/s00167-021-06514-x PMC8800884

[43] Yao L , Dungan D , Seeger L . MR imaging of tibial collateral ligament injury: comparison with clinical examination. Skeletal Radiol. 1994;23:521–524. 7824979 10.1007/BF00223082

[44] Zantop T , Lenschow S , Lemburg T , Weimann A , Petersen W . Soft‐tissue graft fixation in posterior cruciate ligament reconstruction: evaluation of the effect of tibial insertion site on joint kinematics and in situ forces using a robotic/UFS testing system. Arch Orthop Trauma Surg. 2004;124:614–620. 15372279 10.1007/s00402-004-0741-y

